# Piperine alleviates nonalcoholic steatohepatitis by inhibiting NF-κB-mediated hepatocyte pyroptosis

**DOI:** 10.1371/journal.pone.0301133

**Published:** 2024-03-28

**Authors:** Suye Ran, Lingyu Song, Hong Yang, Jiangnan Yu, Yunhuan Zhen, Qi Liu

**Affiliations:** 1 Department of Gastroenterology, The Affiliated Hospital of Guizhou Medical University, Guizhou Medical University, Guiyang, China; 2 Department of Colorectal Surgery, The Affiliated Hospital of Guizhou Medical University, Guiyang, China; Nihon University School of Medicine, JAPAN

## Abstract

**Purpose:**

Nonalcoholic steatohepatitis (NASH) is the progressive form of nonalcoholic fatty liver disease (NAFLD), which has a high risk of cirrhosis, liver failure, and hepatocellular carcinoma. Piperine (Pip) is an extract of plants with powerful anti-inflammatory effects, however, the function of Pip in NASH remains elusive. Here, we aim to explore the role of Pip in NASH and to find the possible mechanisms.

**Methods:**

Methionine and choline-deficient (MCD) diets were used to induce steatohepatitis, methionine- and choline-sufficient (MCS) diets were used as the control. After Pip treatment, H&E staining, Oil Red O staining, hepatic triglyceride (TG) content and F4/80 expression were performed to analysis liver steatosis and inflammation; Masson’s staining, COL1A1 and α-SMA were detected liver fibrosis. Lipopolysaccharide (LPS) -treated AML12 cells were used to as the cell model to induce pyroptosis. Then, pyroptosis-related proteins, IL-1β and LDH release were detected *in vivo* and *in vitro*. Finally, NF-κB inhibitor, BAY11-7082, was used to further demonstrate the mechanism of Pip in NASH.

**Results:**

The study found that Pip alleviated liver steatosis, inflammation, hepatocyte injury, and fibrosis in mice fed with MCD diets. Moreover, the pyroptosis markers (NLRP3, ASC, caspase-1 p20, and GSDMD), IL-1β and LDH release were decreased by Pip treatment. NF-κB activation was suppressed by Pip treatment and pyroptosis-related proteins were down regulated by BAY11-7082.

**Conclusion:**

Pip ameliorates NASH progression, and the therapeutical effect was associated with inhibition of hepatocyte pyroptosis induced by NF-κB.

## Introduction

NAFLD is the most common chronic liver disease, and its prevalence is rapidly increasing due to improvements in life conditions [1]. NAFLD includes a wide spectrum of liver damage, ranging from lipid accumulation in the liver to NASH, characterized by liver steatosis, inflammation, hepatocellular injury, and different degrees of fibrosis. Several studies have shown that individuals with NASH are at high risk of cirrhosis, liver failure, and hepatocellular carcinoma [2], and 10%–25% of individuals with NAFLD progress to NASH [3]. Unfortunately, there are no approved regimens for NASH treatment thus far. Hence, understanding the mechanisms responsible for switching from benign nonalcoholic fatty liver to NASH represents a major clinical challenge.

According to recent studies, liver inflammation is the key factor that fuels the transition from nonalcoholic fatty liver to NASH [4]. Pyroptosis, the most recent identified proinflammatory form of programmed cell death, is downstream of NLRP3 inflammasome activation. In this process, active caspase-1 cleaves GSDMD, a pyroptosis executor that triggers the release of IL-1β and IL-18, thereby activating the inflammatory response [5]. High expression of NLRP3 has been reported in NASH patients and mouse models [6]. Inhibition of NLRP3 results in alleviation of hepatocyte pyroptosis, liver inflammation, and fibrosis [7]. Consistently, Xu et al. showed that MCD-fed Gsdmd-/- mice are protected from steatohepatitis and fibrotic symptoms [8]. These studies suggest that hepatocyte pyroptosis plays an important role in NASH.

Pip is a major plant alkaloid derived from black pepper that has been reported to possess anti-inflammatory properties [9]. Many studies have found that Pip improves chronic inflammatory diseases, such as inflammatory bowel disease, gastritis, and osteoarthritis [10–12]. Moreover, according to many previous reports, Pip plays a protective role in many diseases, including diabetic nephropathy, Staphylococcus aureus endometritis, and lipopolysaccharide-induced acute lung injury, by inhibiting NF-κB activation [13]. However, the role of Pip in NASH remains unexplored.

In the present study, we investigated the effect of Pip on MCD-induced NASH and the potential mechanism involved. We verified that the NASH phenotype of the liver in MCD mice was improved after Pip treatment. Moreover, hepatocyte pyroptosis induced by MCD or LPS was suppressed by Pip *in vivo* and *in vitro*, and this process was associated with inhibition of the NF-κB transcription factor.

## Materials and methods

### Animals

C57BL6/J male mice (6 weeks old) were purchased from Chongqing city, China and kept in the Laboratory Animal Centre of Guizhou Medical University with a 12-hour light-dark cycle and drinking water at 22°C. MCS diets and MCD diets used in this experiment were purchased from Mediscience Ltd. (Jiangsu, China). Pip was obtained from Aladdin and dissolved in dimethylsulfoxide (DMSO) at a concentration of 100 mg/ml.

Mice were randomly divided into three groups (n = 8 for each group). After two weeks of adjustable feeding, MCD diets were used to induce steatohepatitis, and MCS diets were given as the control. To investigate the protective effect of Pip, the mice in the MCD+Pip group were administered Pip at a dose of 10 mg/kg by intraperitoneal injection, and the mice were fed MCD diets. The corresponding amount of DMSO was administered to the mice in the MCS and MCD groups once a day. After 4 weeks of treatment, mice were anesthetized with intraperitoneal injection of 1.25% Tribromoethanol (Nanjing Aibei Biotechnology Co., LTD) (0.2 ml/10 g body weight) [14,15], then sacrificed by cervical dislocation, with the liver isolated and peripheral blood drawn and try to minimize suffering of the experimental mice in the course of operation. Liver tissues were weighed, frozen in liquid nitrogen, and immediately stored at -80°C. Serum samples were collected and immediately stored at -80°C. All animal experiments were approved by the Guizhou Medical University Institutional Animal Care and Use Committee (No. 2000639).

### Cell culture

The mouse AML12 cell line established from normal hepatocytes was purchased from Zhong Qiao Xin Zhou Biotechnology Co., Ltd. and cultured in the corresponding complete medium containing 10% FBS, 1% liquid media supplement, 40 ng/ml dexamethasone, and 1% penicillin/streptomycin (Zhong Qiao Xin Zhou Biotechnology Co., Ltd., China). All cells were maintained at 37°C in 5% CO_2_ and 95% air.

### Histopathological analysis

The livers from mice were fixed with 4% paraformaldehyde, embedded in paraffin, sectioned (4 μm thick), and stained with H&E staining. The histological score for the NAFLD activity score (NAS) was assessed according to the Kleiner/Brunt criteria, and it is used to evaluate steatosis and inflammation [16]. The histological characteristics of steatosis were scored as follows: steatosis, 0–3; hepatocellular ballooning, 0–2; lobular inflammation, 0–3; and fibrosis, 0–4 [16,17]. Each item in NAS score includes the following (point/s):

(a) Hepatocyte steatosis percentage: 0 (<5%); 1 (5%-33%); 2 (34%-66%); 3 (>66%);

(b) Hepatocellular ballooning (distinct swelling of hepatocytes, pale cytoplasm, some cells appearing ballooned):

0: None (normal hepatocyte morphology, sharp angles, cells appear pink);

1: Rare (clusters of liver cells with cytoplasmic vacuolation, appearing mesh-like, cells vary in size and shape);

2: Common (in addition to the above mentioned pathology, there is one hepatocellular ballooning in the field of view and at least one other balloon-like cell that is twice the size of the former);

(c) Lobular inflammation, counting inflammatory foci at 200X magnification: 0: None; 1: (<2 foci); 2: (2–4 foci); 3: (>4 foci);

(d) Liver fibrosis (0–4):

0: No fibrosis;

1: Fibrosis limited to the portal vein area or perisinusoidal;

2: Perisinusoidal fibrosis and periportal fibrosis;

3: Bridging fibrosis (fibrosis between central veins, central veins and portal tracts and between portal tracts);

4: Highly suspicious or diagnosed cirrhosis, including NASH-related cirrhosis and cryptogenic cirrhosis (steatosis and inflammation decrease as fibrosis progresses). The final NAS was calculated as the sum of each histological score with the following scale: NAS <3 points indicated that NASH was excluded; NAS >4 points indicated a diagnosis of NASH; and NAS in between the above scores indicated that NASH was possible [18]. Masson’s trichrome staining was used to evaluate the degree of collagen deposition according to the manufacturer’s protocol (Solarbio, China). Another portion of the liver tissue was embedded in OCT medium, sectioned (10 μm thick), and stained with an Oil Red O Stain Kit (Nanjing Jiancheng Bioengineering Institute, China) to evaluate lipid accumulation in the mouse liver according to the manufacturer’s protocol. After staining, images were acquired using an Olympus microscope.

### Biochemical analysis

The TG content in livers was determined with a triglyceride assay kit (Applygen Technologies, Beijing, China) at 550 nm. Alanine aminotransferase (ALT), aspartate aminotransferase (AST), and lactate dehydrogenase (LDH) levels were detected using ALT, AST, and LDH assay kits, respectively (Nanjing Jiancheng Bioengineering Institute, China), according to the manufacturer’s protocol. IL-1β levels in mouse serum and cell culture supernatant were detected with a mouse IL-β ELISA kit (Elabscience) at 450 nm.

### Quantitative real-time PCR (qRT-PCR)

According to our previous studies [19], total RNA was extracted from liver tissue and lysed in TRIzol reagent (Ambition). Samples (1 μg of RNA) were reverse transcribed to synthesize cDNA using a cDNA Reverse Transcription Kit (Takara, Japan). qRT-PCR was conducted with SYBR Premix Ex Taq (Takara, Japan) and then analysed with the StepOne Real-Time PCR System (Applied Biosystem). The following thermocycler protocol was utilized for qRT-PCR: predenaturation at 95°C for 2 min; and 40 cycles of denaturation at 95°C for 10 s, annealing at 60°C for 30 s, and extension at 72°C for 15 s. The relative expression of each mRNA was calculated with the 2^−ΔΔCt^ method, and *beta-actin (β-actin)* was used as the reference gene. All reactions were performed in duplicate, and the mean values were used for analysis. The sequences of the primers used for qRT-PCR are listed in Table 1.

**Table 1:** Primers used for qRT-PCR analysis.

Gene name	Forward primer sequence	Reverse primer sequence
ACC1	CGAAGGGCTTACATTGCCTA	GGATGTTCCCTCTGTTTGGA
SREBP-1c	CACTTCTGGAGACATCGCAAAC	ATGGTAGACAACAGCCGCATC
SCD1	TGTCTCGGTGTGTGTCGGAGT	TGTACCACTACCTGCCTGCATG
CPT-1A	TCAAGCCAGACGAAGAACATC	TGGTAGGAGAGCAGCACCTT
ACOX1	TTCTCAACAGCCCAACTGTG	GGCATGTAACCCGTAGCACT
IL-1β	CCAGGATGAGGACATGAGCA	CGGAGCCTGTAGTGCAGTTG
TNF-α	ACTGGCAGAAGAGGCACTCC	GCCACAAGCAGGAATGAGAA
β-actin	TCATCACTATTGGCAACGAGC	AACAGTCCGCCTAGAAGCAC

### Immunohistochemistry analysis (IHC)

For immunohistochemistry analysis, the paraffin sections were immersed in 3% H_2_O_2_ for 10 min to block endogenous peroxidases, and they were then blocked with 5% BSA for 30 min and incubated with primary antibodies against F4/80 (Abcam, 1:100), CoL1A1 (Abcam, 1:100), and NF-κB p65 (Santa Cruz, 1:50) overnight at 4°C. The sections were then incubated with the appropriate secondary antibody (ZSGB-BIO) for 2 h and treated with diaminobenzidine (Solarbio, China) for approximately 5 min until the desired colour appeared. All sections were subsequently counterstained with haematoxylin for 30 s, dehydrated, and mounted with neutral gum. Negative control groups were subjected to a similar method, except that the primary antibodies were incubated. Images were acquired using an Olympus optical microscope. Histological assessment was performed by two independent pathologists who were blinded to the experiments. The stained areas of histological section were assessed in 5 random fields per mouse using Image-Pro Plus 6.0 software.

### Western blot analysis

The western blot protocol was conducted as previously described [20]. The protein was from liver tissues, and AML12 cells were lysed with RIPA buffer (Beyotime Biotechnology, China), containing proteinase inhibitor cocktail (Roche Applied Science, USA) and phosphatase inhibitors (Solarbio Life Science, China), for 30 min at 4°C. Protein concentrations were determined using a BCA kit (Beyotime Biotechnology, China). After denaturation and sonication, the proteins were loaded on SDS-polyacrylamide gel electrophoresis gels for electrophoresis and transferred to PVDF membranes. The membranes were then incubated with the appropriate primary antibodies against NLRP3 (ABclonal, 1:500), GSDMD (Santa Cruz, 1:1000), caspase-1 p20 (Santa Cruz, 1:1000), ASC (Santa Cruz, 1:1000), p-NF-κB p65 (Santa Cruz, 1:1000), and NF-κB p65 (Santa Cruz, 1:1000) overnight at 4°C. HRP-conjugated secondary antibodies were used to visualize protein expression (ZSGB-BIO). Final detection was performed using the GENEsys system, and the protein bands were analysed by ImageJ software. The western blot bands used in this manuscript correspond to the original bands with markers in S1 Raw images.

### Cell viability assay

The 3-(4,5-cimethylthiazol-2-yl)-2,5-diphenyl tetrazolium bromide (MTT) assay was performed to measure cell viability after treatment with different doses of Pip. Briefly, cells were seeded into 96-well plates at a density of 1 × 10^3^ per well and incubated overnight. Cells were then treated with increasing doses of Pip for 24–48 h. Cells were then washed with PBS, and MTT solution (0.5 mg/ml in PBS) was added for another 4 h incubation at 37°C. After removing the MTT solution, cells were washed with PBS, and formazan was dissolved in DMSO. The absorbance was analysed by a spectrophotometer at 490 nm.

### Statistical analysis

All experiments were repeated in triplicate. Statistical analyses were performed by GraphPad Prism 7, and the data are presented as the mean± SEM. One-way ANOVA followed by Tukey’s multiple comparisons test was used for parametric data, and the Kruskal–Wallis test was used if the data were not normally distributed. Differences with *p* values < 0.05 were considered statistically significant. The statistics involved in this study are included in the S1 Data.

## Results

### Pip alleviates liver steatosis and inflammation induced by an MCD diet in mice

**Fig 1:**
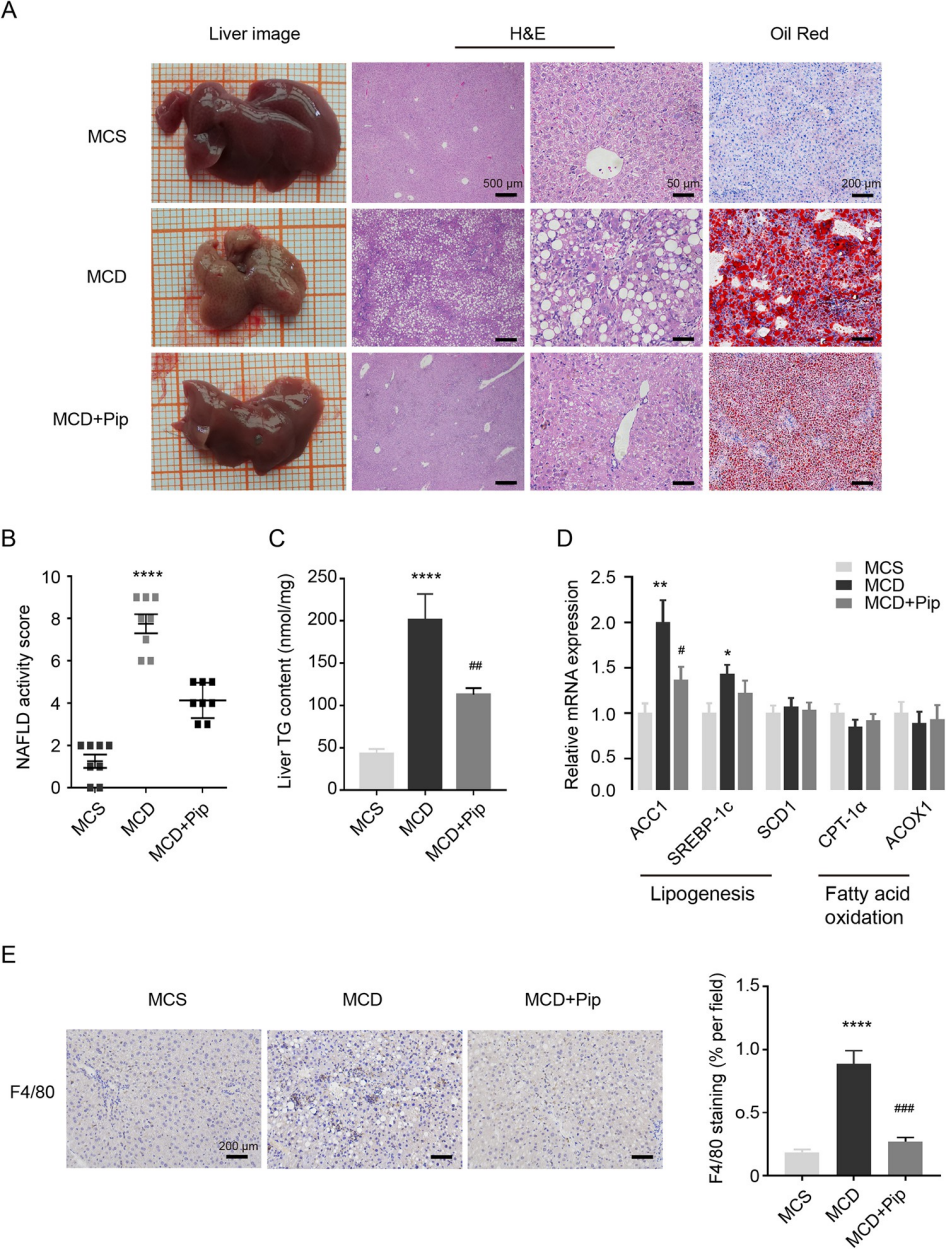
Effects of Pip on hepatic steatosis and inflammation in MCD diet-induced mice. **(A)** Liver appearance was assessed by H&E staining (40x, scale bar = 500 μm; 400x, scale bar = 50 μm) and Oil Red O staining (100x, scale bar = 200 μm) of the three groups of mice. H&E staining was used to evaluate lipid accumulation, the inflammatory response, and ballooning in mouse liver tissue. Oil Red O staining was used to evaluate lipid accumulation in mouse liver tissue. **(B)** Analysis of H&E staining to assess the severity of NAFLD in the mouse model using the histological NAS. **(C)** Liver TG measurement in the three groups of mice after 4 weeks of feeding. **(D)** qRT–PCR was utilized to detect genes related to lipogenesis and fatty acid oxidation. **(E)** IHC was used to detect the expression of the mouse hepatic macrophage biomarker, F4/80. Image-ProPlus 6.0 software was used to evaluate 5 randomly stained areas in each mouse (n = 4) (100x; Scale bar = 200 μm). MCS group, mice were fed an MCS diet with no treatment (control group, MCS); MCD group, mice were fed an MCD diet to induce steatohepatitis (MCD); and MCD+Pip group, mice were fed an MCD diet and treated with Pip (MCD+Pip). MCD vs. Except for IHC, all data are shown as the mean ± SEM (n = 8). Except for the histological NAS analysis using Kruskal-Wallis test, the rest of the data was analyzed using one-way ANOVA. MCS: *p <0.05, **p <0.01, ***p <0.001, and ****p <0.0001; MCD vs. MCD+Pip: ^#^*p* <0.05, ^##^*p* <0.01 and ^###^*p* <0.001.

To investigate the role of Pip in NASH, male C57BL6/J mice were challenged with an MCD diet for 4 weeks to induce steatohepatitis, and littermates of the same age fed an MCS diet were used as the reference group. As expected, mice fed the MCD diet had significantly reduced body weight, liver weight, and body weight/liver weight ratio, whereas there were no significant differences between the MCD group and the MCD+Pip group, except for body weight (Table 2). H&E staining was utilized to assess the severity of NAFLD by NAS, which showed that the MCD+Pip group had alleviated steatosis, inflammatory lesions, and hepatocellular ballooning compared to the MCD group (Fig 1A and 1B).

**Table 2:** General parameters evaluated in mice.

	MCS	MCD	MCD + Pip
Initial body weight (g)	23.35 ± 0.49	23.15 ± 0.43	23.48 ± 0.31
Final body weight (g)	26.01 ± 0.55	15.39 ± 0.37****	17.21 ± 0.35^#^
Liver weight (g)	1.22 ± 0.05	0.62 ± 0.02****	0.67 ± 0.05
Liver/Body weight (%)	4.71 ± 0.24	4.06 ± 0.23***	3.38 ± 0.12
Food intake (g/day/mouse)	3.48 ± 0.40	3.28 ± 0.37	3.44 ± 0.35

MCS group, mice were fed an MCS diet with no treatment (control group, MCS); MCD group, mice were fed an MCD diet to induce steatohepatitis (MCD); and MCD+Pip group, mice were fed an MCD diet and treated with Pip (MCD+Pip). All data are expressed as the mean ± SEM (n = 8). Data were analyzed using one-way ANOVA. MCD vs. MCS: ***P <0.001; ****P <0.0001; MCD vs. MCD + Pip group: ^#^*P* <0.05.

Oil Red O staining showed that the MCD-induced accumulation of hepatic lipid droplets was reduced by Pip treatment (Fig 1A). TG accumulation is the major contributor to liver steatosis [21], and we also found that the hepatic TG content showed similar results to those of Oil Red O staining (Fig 1C). Because TG metabolism mainly includes lipogenesis and fatty acid oxidation, we further evaluated the expression of genes involved in lipid metabolism. We found that Pip downregulated lipogenesis genes, such as *acetyl-CoA carboxylase 1 (ACC1)* and *sterol regulatory element-binding protein-1c (SREBP-1c)*, rather than lipolysis genes (Fig 1D). Therefore, these results suggested that the effect of Pip on hepatic lipid accumulation is associated with reduced lipogenesis in the liver via modulating lipogenic gene expression of *ACC1* and *SREBP-1c*.

Hepatic macrophages play a central role in the inflammation of NASH pathological processes [22]. To investigate the inflammatory status, the expression of F4/80, a macrophage biomarker, was detected by IHC staining, and we found that the MCD diet-induced F4/80 expression was downregulated by Pip treatment (Fig 1E). Taken together, these results suggested that Pip alleviates liver steatosis and inflammation induced by the MCD diet in mice.

### Pip ameliorates MCD diet-induced liver injury and fibrosis

**Fig 2:**
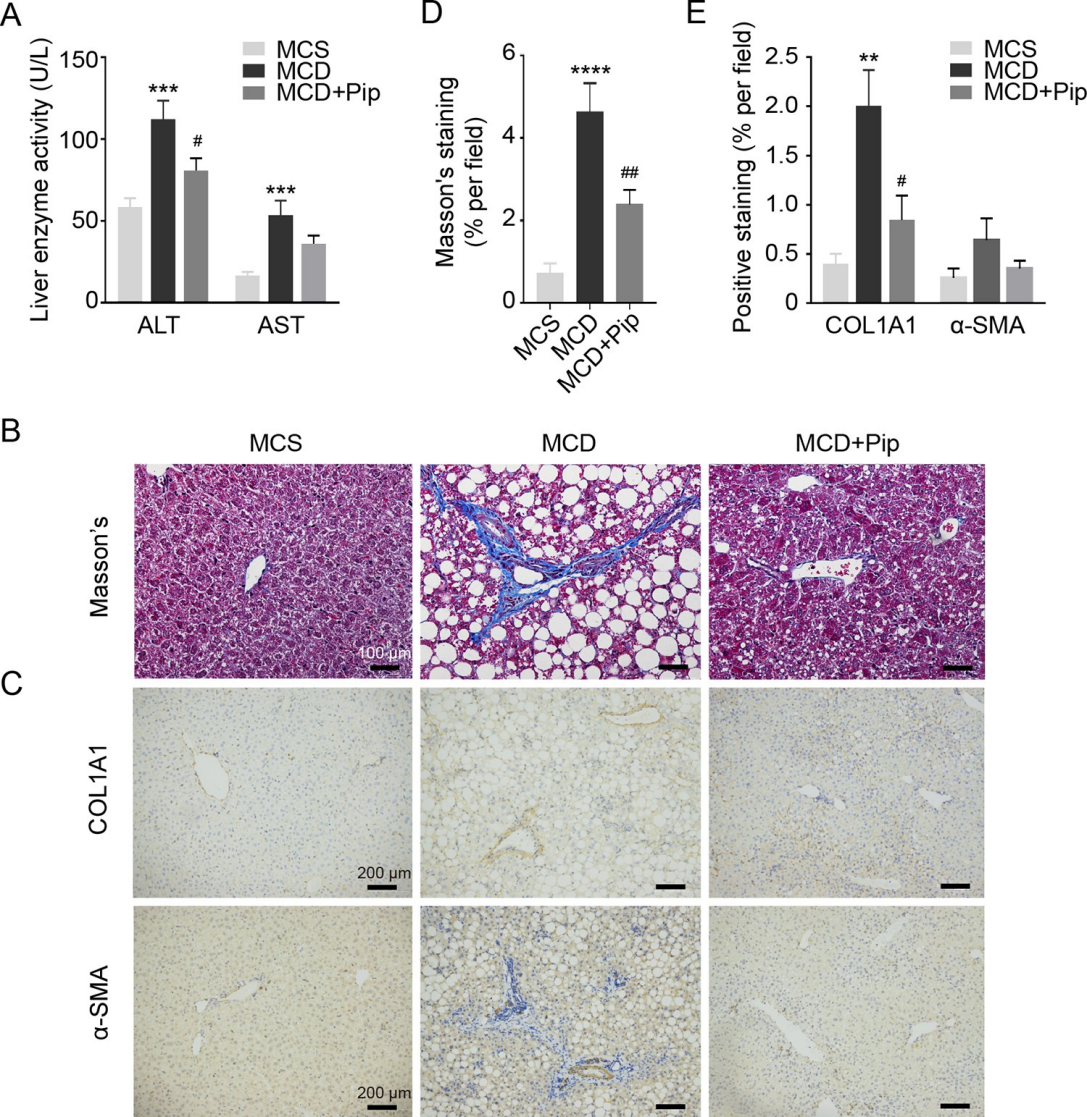
Pip ameliorates hepatic injury and fibrosis in MCD diet-induced mice. **(A)** Serum ALT and AST levels in the three groups of mice. MCS group, mice were fed an MCS diet with no treatment (control group, MCS); MCD group, mice were fed an MCD diet to induce steatohepatitis (MCD); and MCD+Pip group, mice were fed an MCD diet and treated with Pip (MCD+Pip). **(B, D)** Images of Masson’s trichrome staining of mouse liver tissue (200x, Scale bar = 100 μm) were used to assess the severity of liver fibrosis in each group of mice. The positive area of Masson’s trichrome staining was calculated by ImageJ software. **(C)** IHC was used to evaluate the expression of the biomarkers of fibrosis, COL1A1 and α-SMA (100x, scale bar = 200 μm) (n = 4). **(E)** Software was used to calculate the positive area of staining in the histopathological mouse liver sections. All data are shown as the mean ± SEM (n = 8), which was analyzed using one-way ANOVA, except when indicated otherwise. MCD vs. MCS: **p <0.01, ***p <0.001, and ****p <0.0001. MCD vs. MCD+Pip: ^#^*p* <0.05 and ^##^*p* <0.01.

We next investigated the impact of Pip on liver injury. Compared to the MCS group, the plasma levels of ALT and AST were significantly elevated in the MCD group. However, Pip significantly decreased the plasma levels of ALT, and Pip resulted in a decreasing trend of AST levels in the plasma (Fig 2A). Moreover, Masson’s trichrome staining and IHC assays were used to evaluate the degree of fibrosis. We found that collagen deposition was alleviated in the MCD+Pip group compared to the MCD group (Fig 2B and 2D), and the biomarkers of fibrosis, COL1A1 and α-SMA, also presented results similar to those of Masson’s trichome staining (Fig 2C and 2E). These results suggested that Pip ameliorates MCD diet-induced liver injury and fibrosis.

### Pip inhibits NLRP3 inflammasome activation and pyroptosis in mouse livers and AML12 hepatocytes

**Fig 3:**
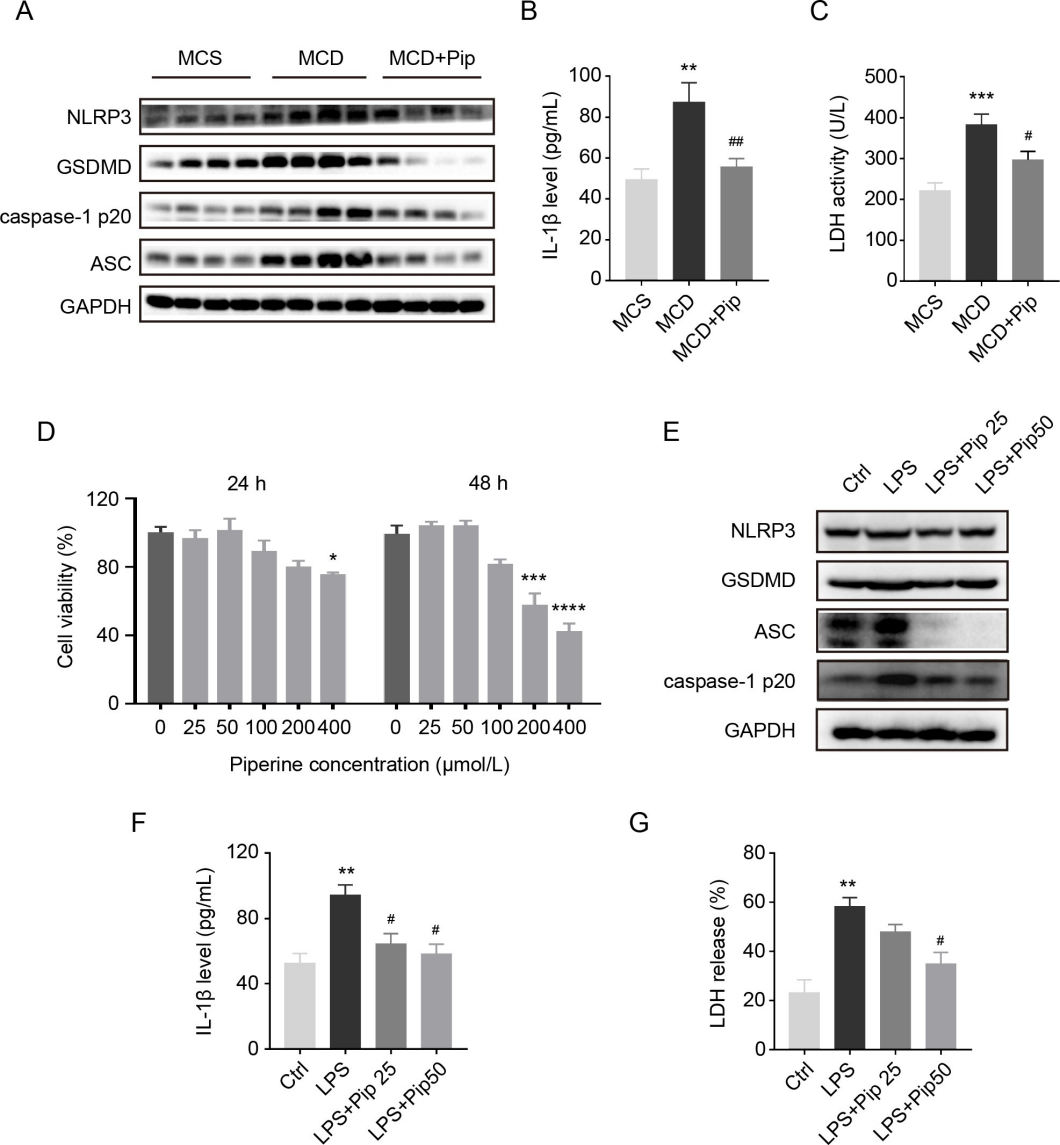
Confirmation of the inhibition of hepatocyte pyroptosis by Pip *in vivo* and *in vitro*. **(A)** Western blot was used to analyse the protein expression of the hepatocyte pyroptosis markers, NLRP3, GSDMD, caspase-1 p20, and ASC, in the three groups of mice. GAPDH was used as a control, and the experiment was repeated three times. MCS group, mice were fed an MCS diet with no treatment (control group, MCS); MCD group, mice were fed an MCD diet to induce steatohepatitis (MCD); and MCD+Pip group, mice were fed an MCD diet and treated with Pip (MCD+Pip). **(B-C)** Detection of IL-1β, the downstream product of the pyroptosis activation pathway, and LDH in mouse serum. **p <0.01 and *** *p* <0.001. **(D)** An MTT assay was performed to evaluate cell viability at different doses of Pip. The 0 μM group was used as the control group: *p <0.05 (24 h), ***p <0.001, and ****p <0.0001 (48 h). **(E)** The protein expression of the hepatocyte pyroptosis markers, NLRP3, GSDMD, caspase-1 p20, and ASC, in the LPS-induced AML 12 cell line was detected by Western blot analysis. GAPDH was used as the internal control. **(F-G)** Detection of IL-1β and LDH in the culture supernatant of the AML12 cell line. LPS-induced AML12 cell group (LPS) compared to the control group (Ctrl): **p <0.01; Piperine-treated group (LPS+Pip) compared to the LPS-treated group: ^#^*p* <0.05. All data are shown as the mean ± SEM (n = 3), which was analyzed using one-way ANOVA.

Hepatocellular pyroptosis plays an important role in the process of steatohepatitis [8,23], and the pyroptosis pathway is mainly triggered by the NLRP3 inflammasome [24]. To investigate the mechanisms underlying the effects of Pip, we observed the process of pyroptosis in a NASH mouse model and AML12 hepatocytes. Increased expression of NLRP3, GSDMD, caspase-1 p20, and ASC was found in the MCD group compared to the MCS group, but these changes in the MCD group were decreased by Pip treatment (Fig 3A). Moreover, the IL-1β level and LDH activity, which were increased by the MCD diet, were both downregulated after Pip treatment (Fig 3B and 3C).

We then further detected pyroptosis biomarkers in AML12 hepatocytes. First, the appropriate dose of Pip in AML12 hepatocytes was identified by the MTT assay (Fig 3D) [25]. Similar results for the pyroptosis markers were obtained in cultured hepatocytes (Fig 3E–3G). Taken together, these results suggested that Pip treatment alleviates hepatocellular pyroptosis and NLRP3 inflammasome activation.

### The inhibitory action of Pip on pyroptosis and NLRP3 inflammasome activation is associated with the NF-κB p65 signalling pathway

**Fig 4:**
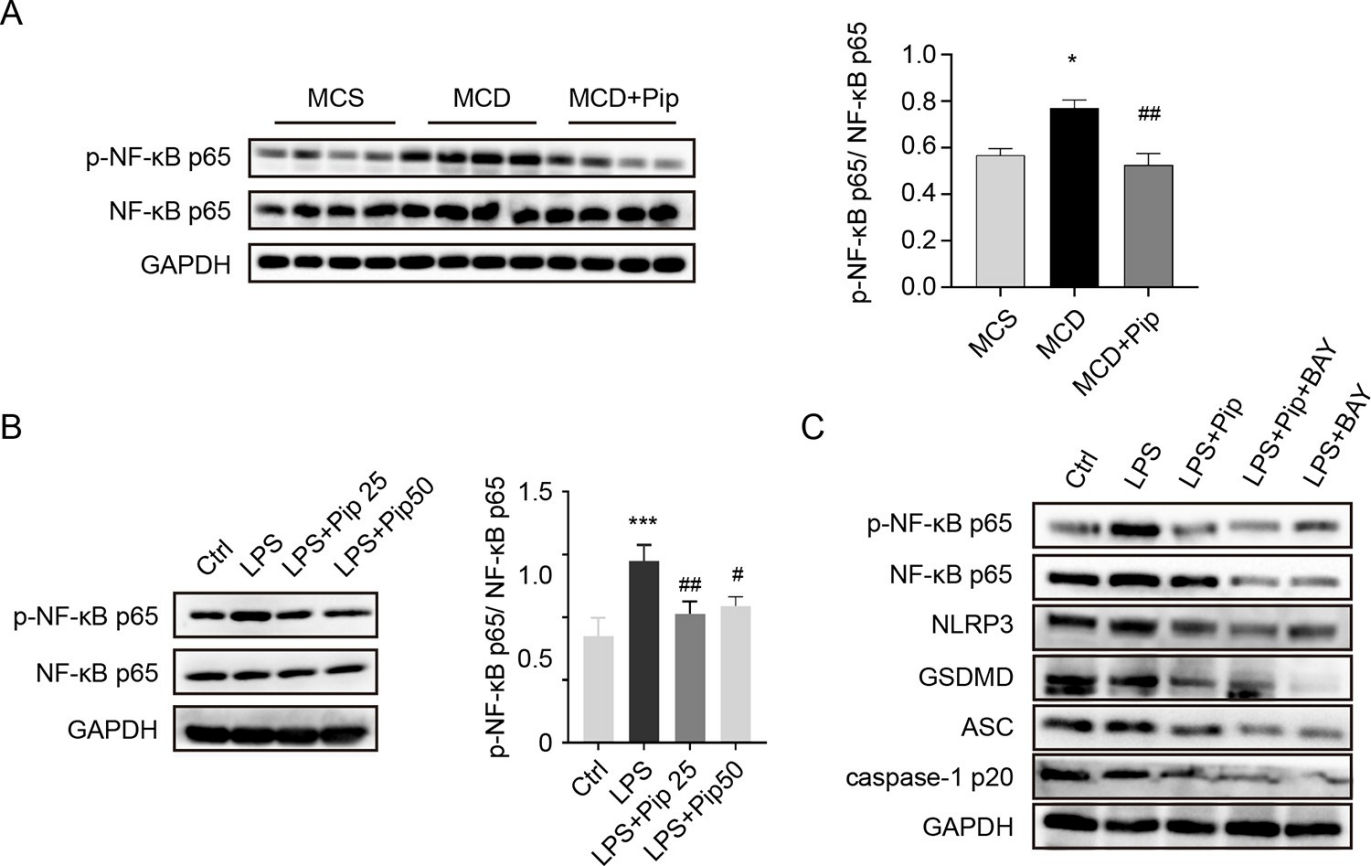
Pip alleviates hepatocellular pyroptosis by downregulating the NF-κB signalling pathway. **(A)** The active protein expression of NF-κB p65 was detected in three groups of mice by Western blot and analysed with ImageJ software. MCS group, mice were fed an MCS diet with no treatment (control group, MCS); MCD group, mice were fed an MCD diet to induce steatohepatitis (MCD); and MCD+Pip group, mice were fed an MCD diet and treated with Pip (MCD+Pip). MCD vs. MCS: *p <0.05; MCD vs. MCD+Pip: ^##^ *p* <0.01 (n = 4). **(B)** Western blot was used to detect the expression of NF-κB p65 activation in the AML12 cell line, and ImageJ software was used to analyse the results. LPS-induced AML12 cell group (LPS) compared to the control group (Ctrl): ***p <0.001; Pip-treated group (LPS+Pip) compared to the LPS-treated group: ^#^*p* <0.05 and ^##^*p* <0.01 (n = 4). **(C)** The expression levels of NF-κB p65 and markers of pyroptosis (NLRP3, ASC, caspase-1 p20, and GSDMD) were evaluated by Western blot analysis in LPS-induced AML12 cells treated with Pip with and without the NF-κB inhibitor, BAY11-7082. GAPDH was used as the internal control. Control group (Ctrl); LPS-induced AML12 cell group (LPS); LPS-induced AML12 cells treated with Pip (LPS+Pip); LPS-induced AML12 cells treated with Pip and NF-κB inhibitor-BAY11-7082 (LPS+Pip+BAY); LPS-induced AML12 cells treated with the NF-κB inhibitor-BAY11-7082 (LPS+BAY). GAPDH was used as a control, and the experiment was repeated three times; All data are shown as the mean ± SEM, and analyzed using one-way ANOVA.

We further explored the mechanism by which Pip inhibits hepatocellular pyroptosis and NLRP3 inflammasome activation by Western blot analysis. The results showed that the expression of p-NF-κB p65 protein, the activated form of NF-κB p65, in the MCD group was significantly increased compared to the MCS group, but p-NF-κB p65 protein expression was decreased after Pip treatment (Fig 4A), which was consistent with the indicators of hepatocyte pyroptosis and inflammation (Fig 3A–3C). In addition, we evaluated the protein levels of pyroptotic indicators in AML12 cells, and the results showed consistent changes (Figs 3E–3G and 4B). To further confirm the effect of NF-κB p65 on pyroptosis, we used the NF-κB inhibitor-BAY11-7082 which can can inhibit the expression and activation of NF-κB p65, to treat LPS-induced AML12 cells. The expression levels of p-NF-κB p65 and the pyroptotic indicators (NLRP3, ASC, caspase-1 p20, and GSDMD) were reduced (Fig 4C), which was consistent with our previous outcomes. The above experimental results indicated that the inhibitory effect of Pip on pyroptosis and NLRP3 inflammasome activation is related to the NF-κB p65 signalling pathway.

## Discussion

NAFLD is now recognized as the most prevalent chronic liver disease worldwide and is thought to be increasing [26,27]. To date, there is no approved pharmacotherapy for this disease [28]. NASH is a progressive form of NAFLD that is considered a risk factor for cirrhosis and even hepatocellular carcinoma (HCC), and it is a major cause of end-stage liver disease and liver transplantation [29]. Therefore, we explored the effects and potential mechanisms of Pip in alleviating NASH in the present study.

In the present study, we successfully induced a NASH mouse model with a MCD diet [30]. After Pip treatment, we observed pathological tissue steatosis, inflammation, fibrosis, and hepatocyte ballooning changes in mice. In addition, Pip significantly reduced the expression of hepatocellular pyroptosis markers (NLRP3, ASC, caspase-1 p20, and GSDMD), and the activation of NF-κB, a regulator of hepatocyte pyroptosis, was also inhibited. We also examined AML12 hepatocytes induced by LPS, which resulted in consistent changes after treatment with Pip. Together, these data showed that Pip attenuates NASH.

Pyroptosis, also known as cell inflammatory necrosis, is a type of programmed cell death that plays an important role in cell growth and maintenance of normal tissue function [31,32]. Therefore, the activation or inhibition of the pyroptotic pathway may have an impact on the development and outcome of the disease. Previous studies have shown that hepatocyte pyroptosis plays an important role in the occurrence and development of NASH [6,33]. In the present study, the H&E and Oil Red O staining of MCD mice treated with Pip showed a reduced liver steatosis, inflammation, and hepatocyte ballooning. In addition, mouse plasma levels of liver injury indices (ALT and AST) showed a decrease after Pip treatment of MCD mice. Masson’s trichrome staining and IHC detection showed that collagen deposition was reduced after Pip treatment. These data suggested that Pip attenuates nonalcoholic steatohepatitis. To further understand the underlying mechanisms of this process, we investigated the components at the molecular level. After treatment with Pip, the expression of pyroptotic indicators (NLRP3, ASC, caspase-1 p20, and GSDMD) was reduced in MCD mice and LPS-induced AML12 cells. Previous studies have shown that the activation of the caspase family plays an important role in the process of pyroptosis, especially classical caspase-1 inflammasome activation [34]. The NLRP3 inflammasome is a member of the NOD-like receptor family, and it combines with apoptosis-associated protein containing a CARD, which further binds with the caspase-1 cysteine protease and forms a complex inflammasome that activates caspase-1 [35]. The activation of caspase-1 cleaves the gasdermin-N (GSDMD-N) structural domain of GSDMD, which is regarded as an executor of pyroptosis, causing the onset of programmed cell death [7,34,36]. Cleavage of GSDMD promotes IL-1β release without affecting its maturation, and it indirectly activates NF-ĸB signalling pathway and the subsequent recruitment of hepatic macrophages for inflammatory development, thereby affecting NASH [37,38]. In addition, a previous study has shown that NF-ĸB activation is associated with GSDMD-N-induced pyroptosis and is a key mechanism in the pathogenesis of steatohepatitis. GSDMD is mainly involved in the pathogenesis of NASH by regulating hepatic fat metabolism, the inflammatory response, and the amplification cascade of the NF-ĸB pathway [8,32]. In addition to affecting lipid metabolism and the inflammatory response, hepatocyte pyroptosis is also linked to liver fibrosis. In the hepatocyte pyroptosis pathway, NLRP3 activation is detected along with the upregulation of fibrogenesis marker genes and stellate cell activation. Thus, the NLRP3 inflammasome induces hepatic stellate cell (HSC) activation and collagen deposition, leading to hepatic fibrosis [23,24,39], consistent with the results of the present study. The expression of NLRP3, ASC, caspase-1p20, and GSDMD was reduced by Pip treatment, which resulted in the inhibition of NLRP3 activation in pyroptosis and led to improved liver fibrosis. In addition, the decreased expression of ASC, caspase-1 p20, and GSDMD suppressed the hepatocyte pyroptosis pathway, which improved the inflammation, steatosis, and cell damage associated with pyroptosis, thereby alleviating NASH.

To gain a deeper understanding of the mechanism by which Pip inhibits pyroptosis and activates the NLRP3 inflammasome, we evaluated the expression of NF-ĸB by western blot analysis. Compared to the MCD group, the expression of p-NF-κB p65 was significantly reduced in the MCD+Pip group. We treated LPS-induced AML12 cells with the NF-κB inhibitor, BAY11-7082, which resulted in reduced expression pyroptotic markers. In addition, AML12 cells treated with Pip also showed decreased expression of pyroptotic markers. As mentioned in previous studies, NF-κB is a family of dimeric transcription factors that are critical in the regulation of inflammation [40]. Endogenous or exogenous pathogens bind to the toll-like receptor TLR4 to activate NF-κB, and then the NLRP3 inflammasome is activated and participated in the regulation of the pyroptotic pathway [41–43]. When Pip treatment inhibits NF-κB activation, NLRP3 inflammasome activation-mediated pyroptosis is also inhibited, which is consistent with our experimental results. Therefore, the inhibition of hepatocyte pyroptosis and NLRP3 inflammasome activation by Pip is related to the NF-κB p65 pathway. Previous studies have found that Pip can inhibit lipogenesis, mainly by regulating the expression of key adipogenesis genes PPARγ, SREBP-1c, FAS, Fab-4, Scd1, ACC, etc. [44,45], and inhibiting adipocyte differentiation, lipid drome maturation and TG release [46]. In addition, Pip treatment can prevent obesity and improve lipid metabolism by regulating adipose tissue expansion (ATE) related genes Sfrp5, MEST, PTRF/Cavin1 [47]. The mechanism of Pip inhibiting lipogenesis is not fully understood. NF-κB is widely regarded as a classical signaling pathway that regulates inflammation-related mechanisms [48]. When it is activated, the expression of classical pro-inflammatory factors such as IL-6, IL-1 and TNF released downstream of NF-κB will be significantly increased [49,50]. At the same time, relevant literature reports that classic inflammatory factors such as IL-6, IL-1 and TNF play an important regulatory role in the process of lipogenesis. Christian von Loeffelholz et al. found that IL-6 activates the IL-6-STAT3 pathway and stimulates *mIndy* expression by binding to its receptor, which increases the hepatic uptake of citrate and hepatic lipogenesis of circulating citrate *in vivo* [51]. In addition, in an animal experiment, it was shown that IL-1β stimulation induced IRAKM to interact with and phosphorylate the mitochondrial citrate carrier Slc25a1, which promoted IL-1β-induced mitochondrial citrate transport to the cytoplasm and adipocytes to regenerate fat [52]. In a high-fructose diet-induced mouse model, Jelena Todoric et al. showed that the deterioration of the intestinal barrier and subsequent endotoxemia caused by high-fructose intake triggers liver-induced upregulation of the ACC1, FAS and SREBP1 lipid genes for lipogenesis. Furthermore, TNF was a key factor in stimulating fructose-driven lipogenesis in primary hepatocytes cultured *in vitro* [53]. In conclusion, we hypothesize that Pip may affect lipogenesis by affecting NF-κB-mediated inflammation, but the detailed mechanism needs to be further explored and verified in future studies.

In summary, our results suggest that Pip ameliorates hepatic steatosis, inflammation, fibrosis and hepatic injury. It improves NASH progression by inhibiting NF-κB p65 signaling pathway mediated hepatocyte pyrodeath and NLRP3 inflammasome activation. This shows that Pip may be a viable option for the clinical treatment of NASH.

## Supporting information

S1 Raw images (PDF)

S1 Data (XLSX)
