# Supplementary material for: Piperine alleviates nonalcoholic steatohepatitis by inhibiting NF-κB-mediated hepatocyte pyroptosis
Source: PLoS One. 2024 Mar 28;19(3):e0301133. doi: 10.1371/journal.pone.0301133 (PMC10977780; doi:10.1371/journal.pone.0301133)
Supplement: S1 Raw images — (PDF) [file pone.0301133.s001.pdf]

Fig 3

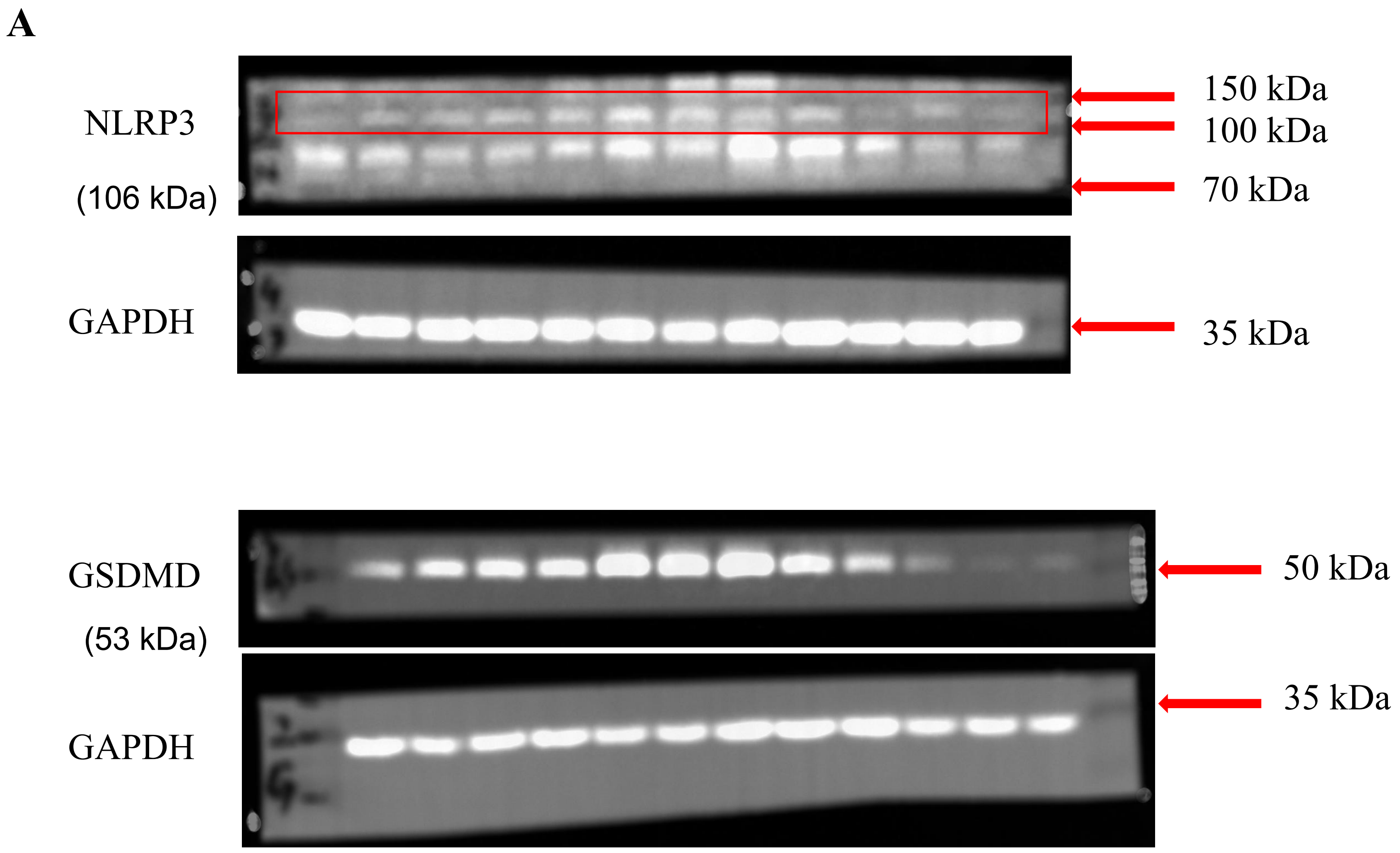
A
NLRP3
(106 kDa)
150 kDa
100 kDa
70 kDa
GAPDH
35 kDa
GSDMD
(53 kDa)
50 kDa
GAPDH
35 kDa

Fig 3

A

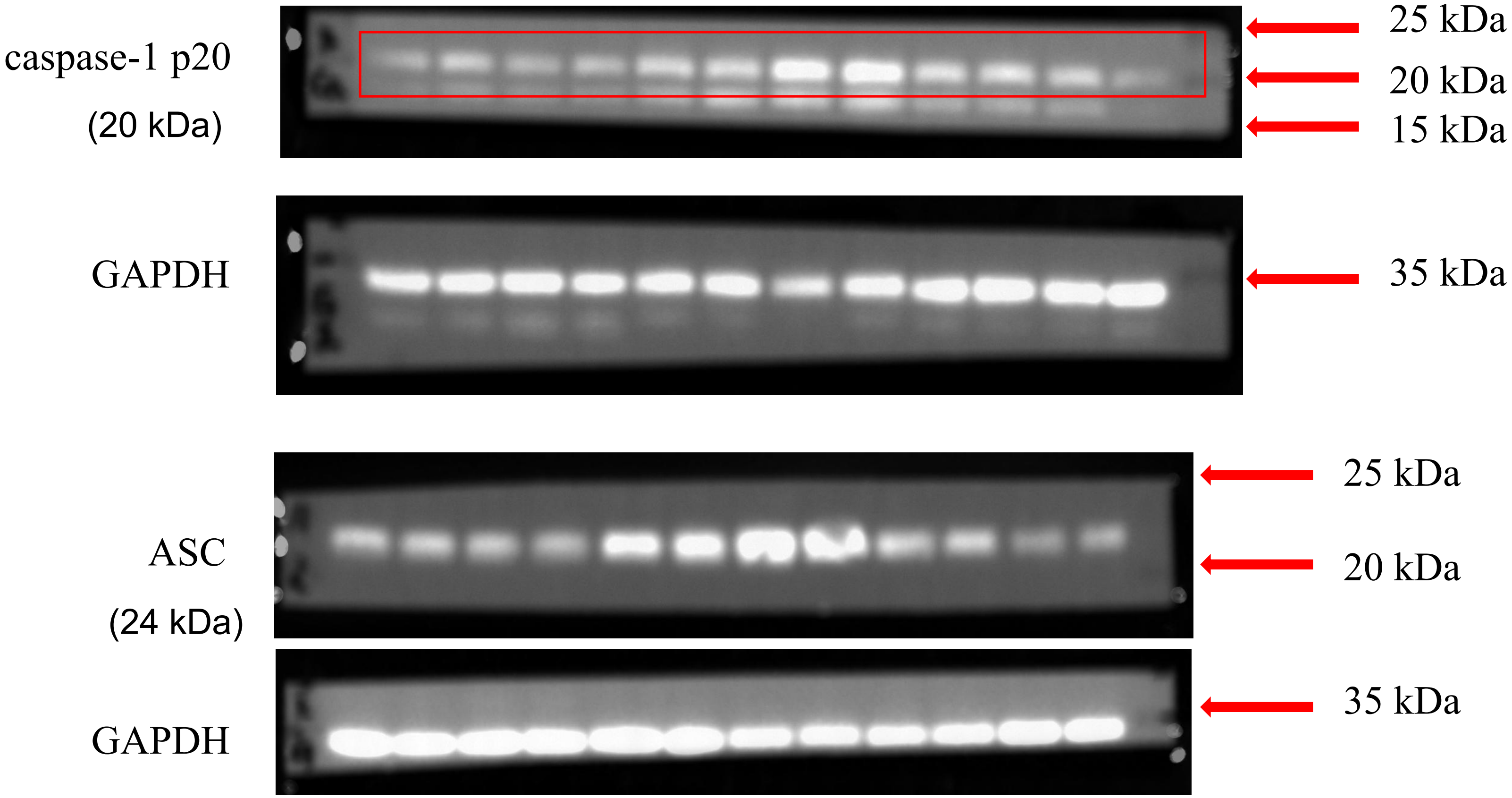
caspase-1 p20
(20 kDa)
25 kDa
20 kDa
15 kDa
GAPDH
35 kDa
ASC
(24 kDa)
25 kDa
20 kDa
GAPDH
35 kDa

Fig 3

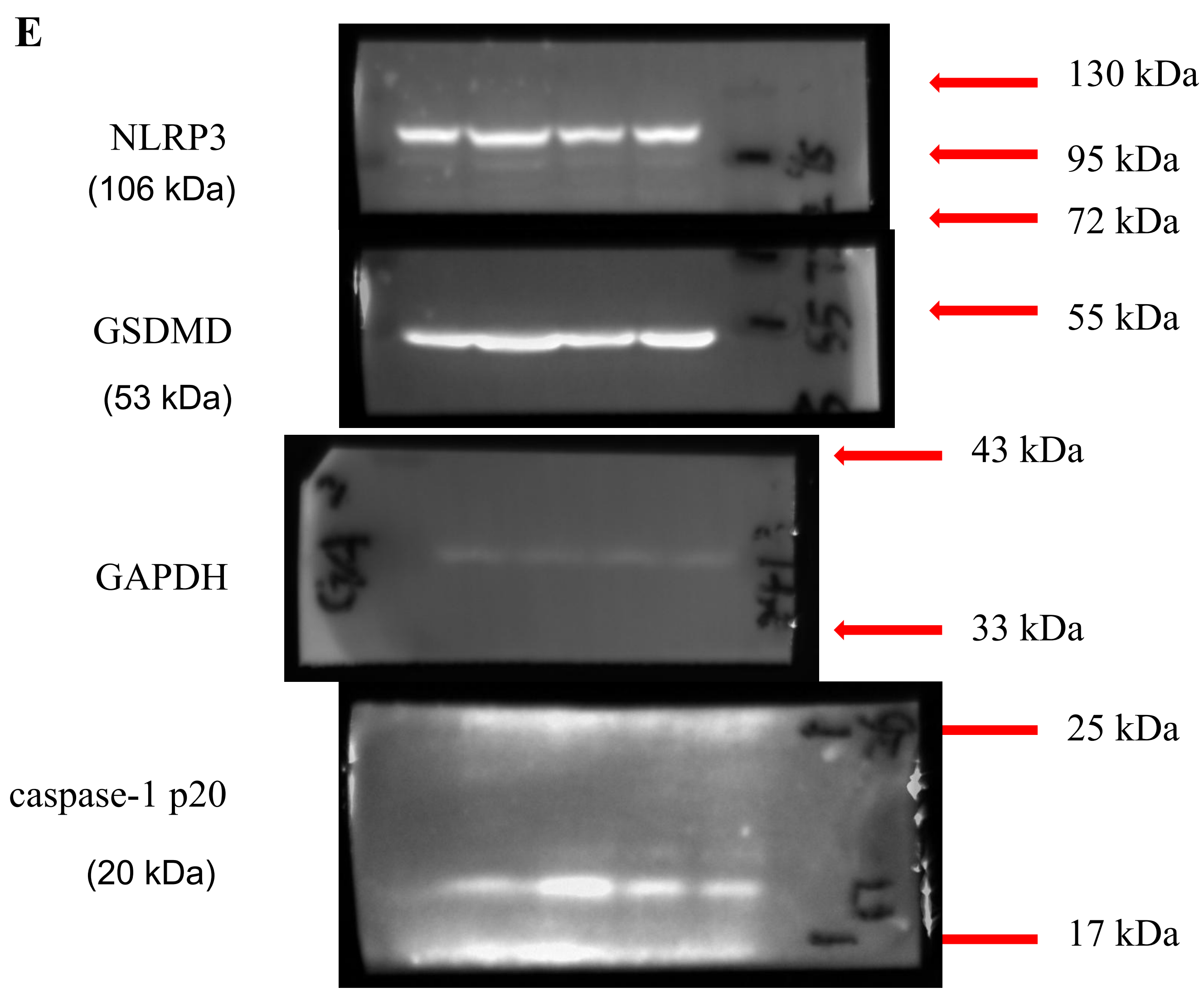
E
NLRP3
(106 kDa)
GSDMD
(53 kDa)
GAPDH
caspase-1 p20
(20 kDa)
130 kDa
95 kDa
72 kDa
55 kDa
43 kDa
33 kDa
25 kDa
17 kDa

Fig 3

E

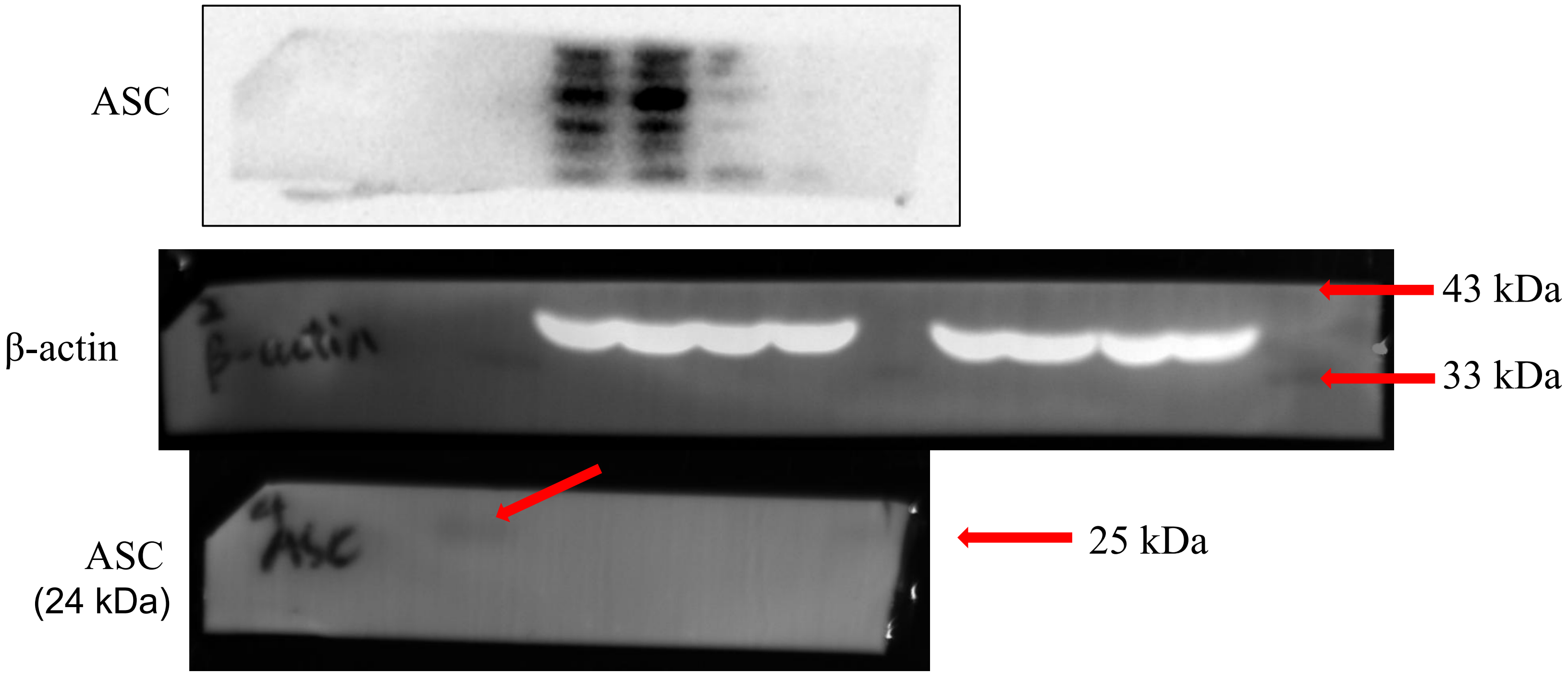

Note: Due to the low molecular weight of ASC, it needs to be turned separately.

Fig 4

A

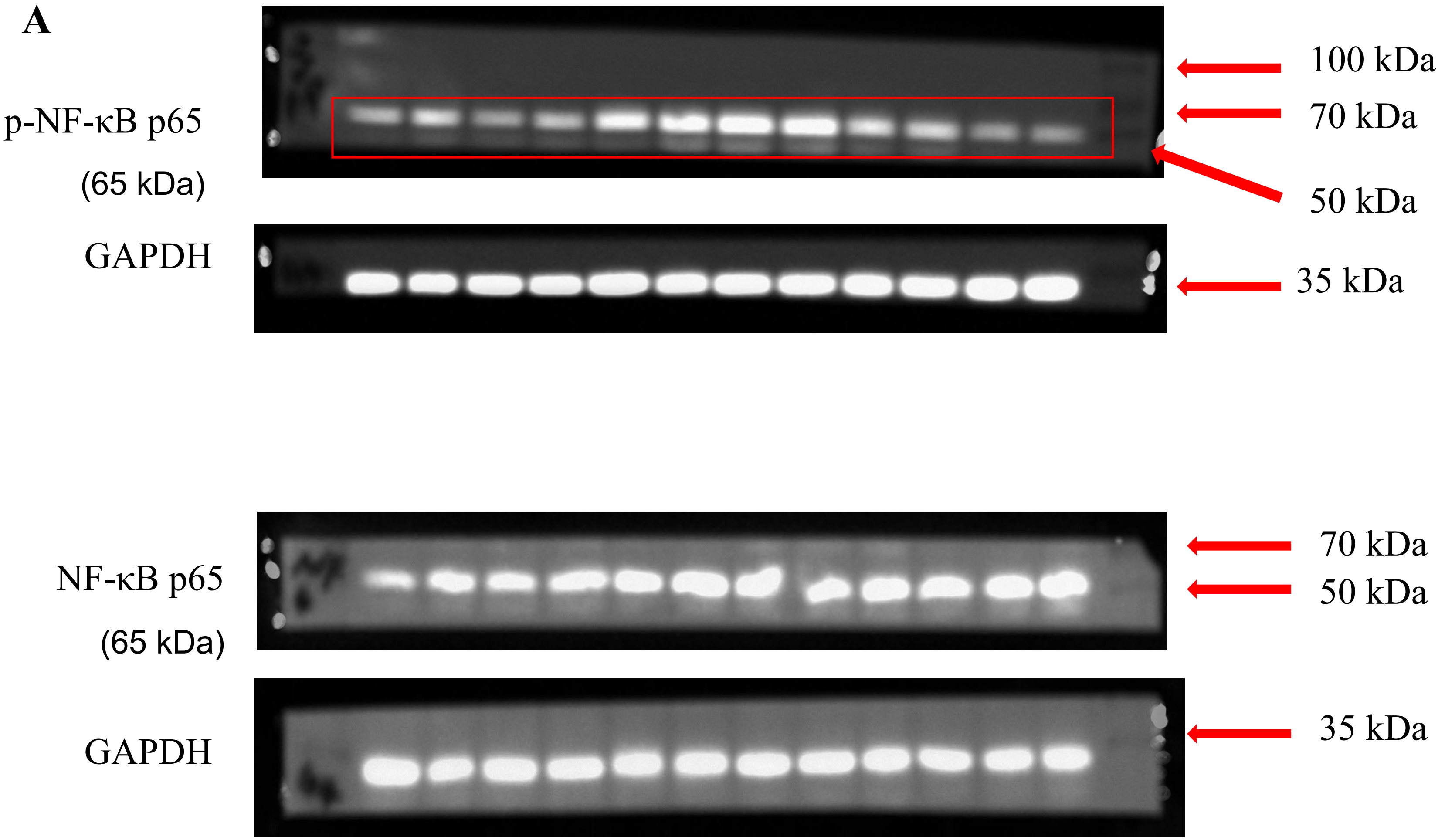

p-NF-κB p65
(65 kDa)
GAPDH
100 kDa
70 kDa
50 kDa
35 kDa
NF-κB p65
(65 kDa)
GAPDH
70 kDa
50 kDa
35 kDa

Fig 4

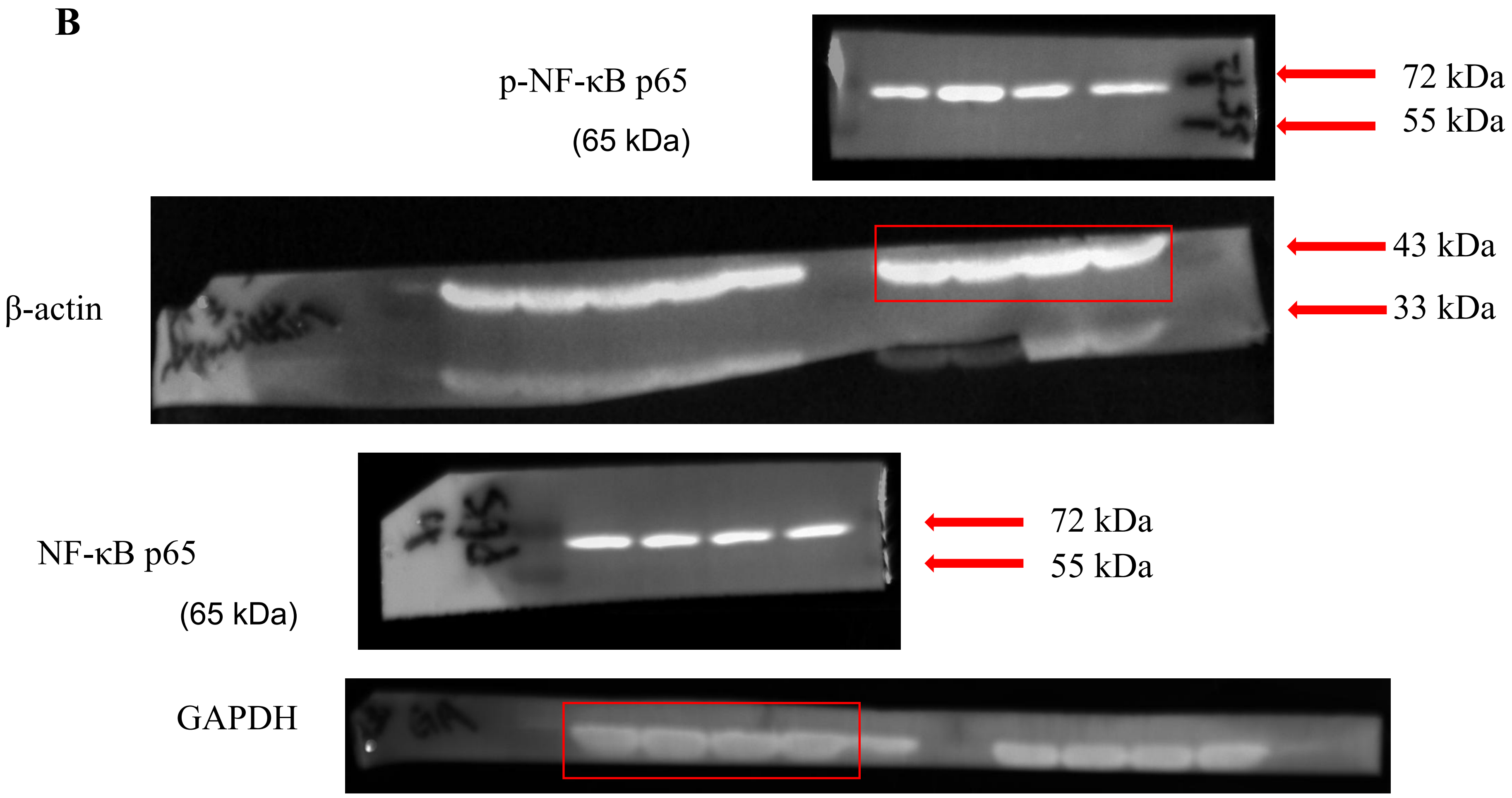
B
p-NF-κB p65
(65 kDa)
72 kDa
55 kDa
β-actin
43 kDa
33 kDa
NF-κB p65
(65 kDa)
72 kDa
55 kDa
GAPDH

Fig 4

C

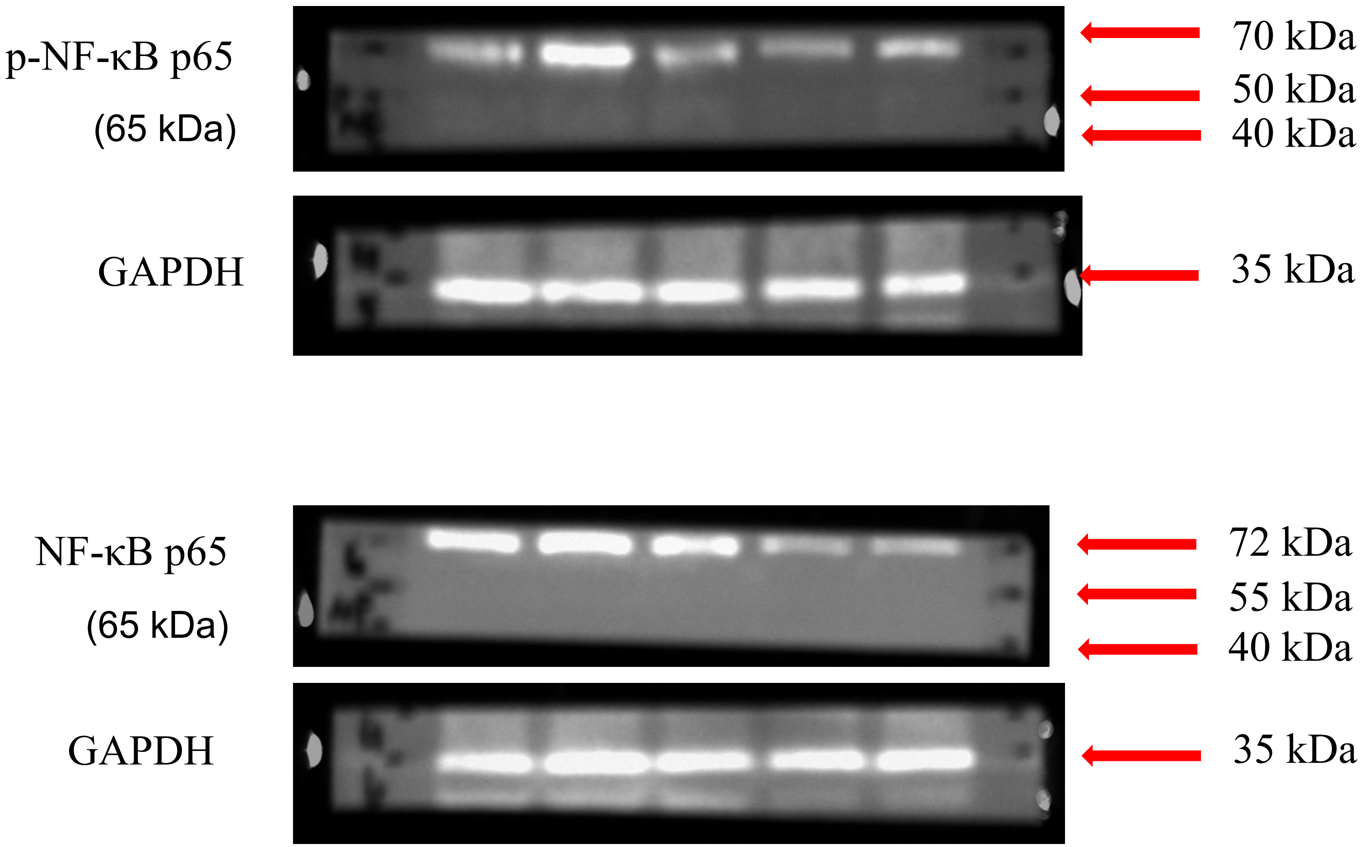

p-NF-κB p65
(65 kDa)
70 kDa
50 kDa
40 kDa
GAPDH
35 kDa
NF-κB p65
(65 kDa)
72 kDa
55 kDa
40 kDa
GAPDH
35 kDa

Fig 4

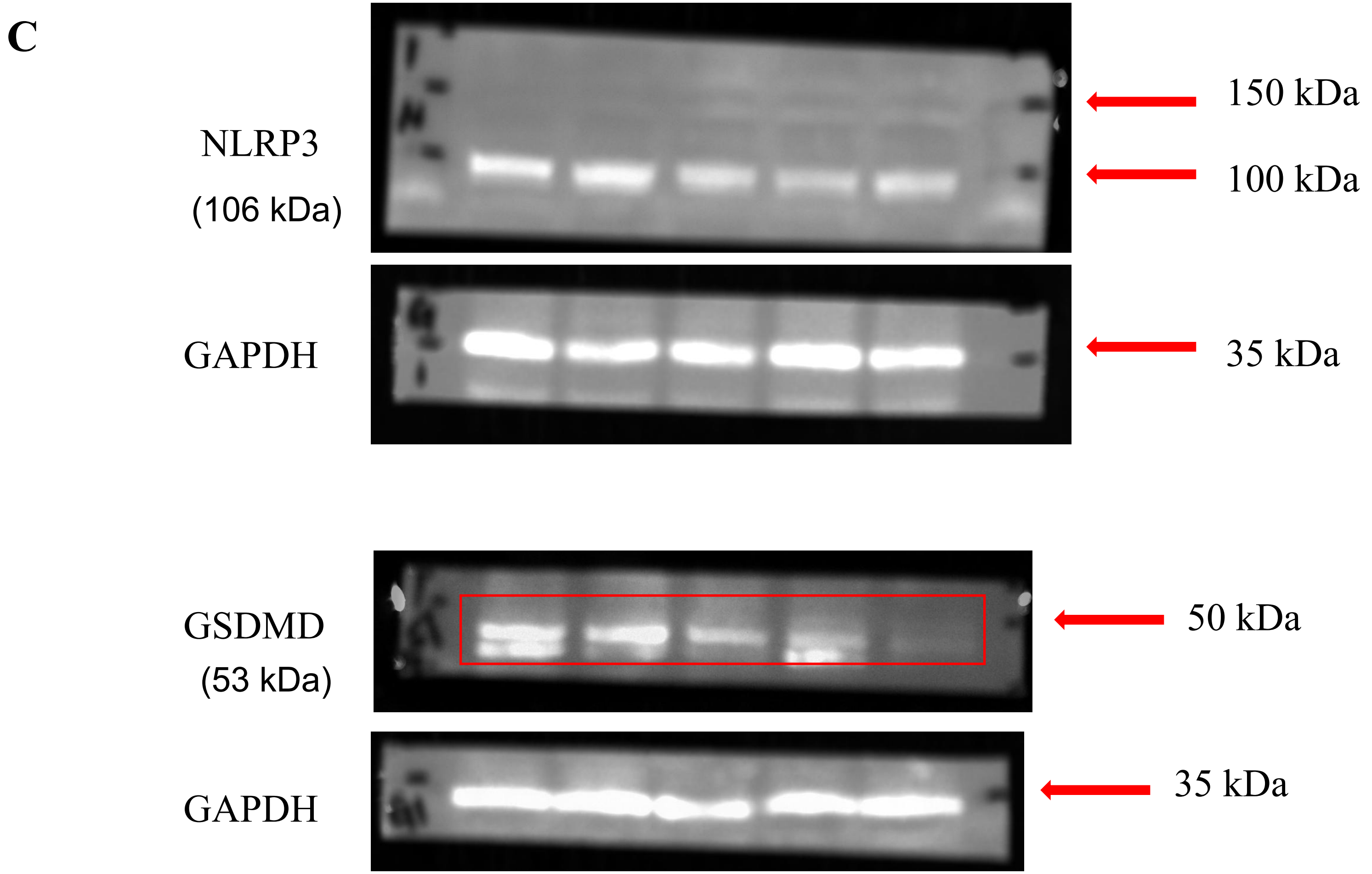
C
NLRP3
(106 kDa)
150 kDa
100 kDa
GAPDH
35 kDa
GSDMD
(53 kDa)
50 kDa
GAPDH
35 kDa

Fig 4

C

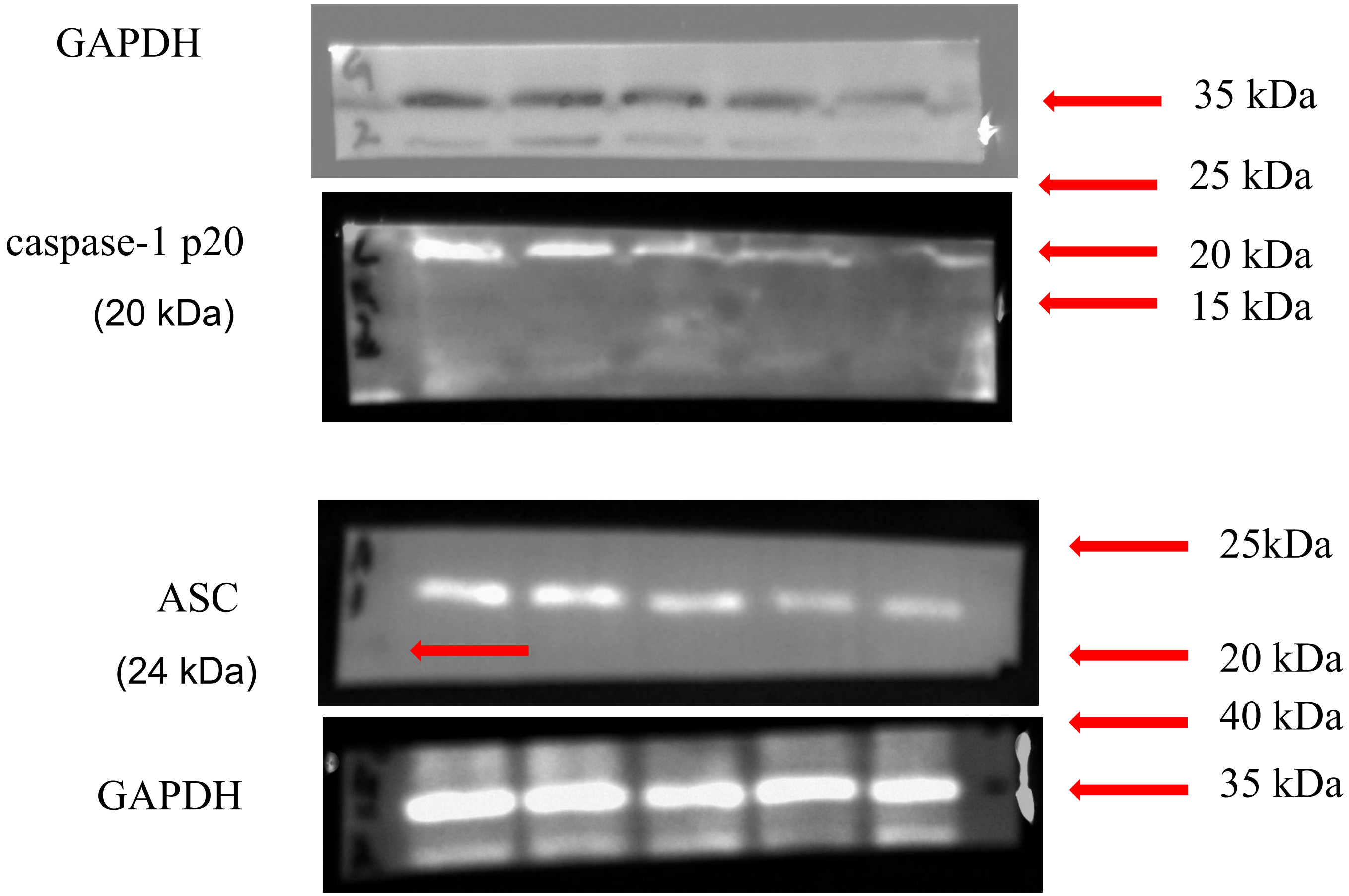
GAPDH
35 kDa
25 kDa
caspase-1 p20
(20 kDa)
20 kDa
15 kDa
ASC
(24 kDa)
25kDa
20 kDa
40 kDa
GAPDH
35 kDa
